# The Army Tuberculosis Examiner

**Published:** 1918-11

**Authors:** 


***The Army Tuberculosis Examiner.*** By C. L. WHEATON (Chicago), Camp Grant, Rockford, Ill. *Journal A. M. A.*, Sept. 7th, 1918.

The author reports his observations as a tuberculosis examiner of recruits or drafted men. In military practice it is of the utmost importance to determine whether a man has tuberculosis or not, and the author goes over the details of the signs by which it can be detected, and the percentages of cases observed, and refers also to the figures reported from France and the statistics of tuberculosis in

Chicago. The methods of eradicating tuberculosis in the army are grouped by him under four headings as follows : "1. Removal of those predisposing factors tending to lower the physiologic resistance of the individual; that is, bad housing, overcrowded, poorly lighted and badly ventilated tenements, inadequate and poor food supply, and unsanitary conditions in general. 2. Removal of foci of infection by hospitalization and segregation of open cases so far as practicable. 3. Effecting a cure of incipient closed cases at a time when this is possible, and before there is softening of lung tissue. 4. Eradication of bovine tuberculosis, and more widespread knowledge of the dangers resulting from the consumption of meat and milk from tuberculous cattle, notwithstanding the fact that the bovine bacillus is less virulent than the human bacillus for man ".

